# Comparison of intranasal and intraperitoneal administration of *Eugenia caryophyllata* (clove) essential oil on spatial memory, anxiety-like behavior and locomotor activity in a pilocarpine-induced status epilepticus rat model

**DOI:** 10.1186/s12906-022-03711-0

**Published:** 2022-08-30

**Authors:** Fatemeh Parvizi, Soraya Mehrabi, Ayeh Naghizadeh, Mohammad Kamalinejad‬, Sepide Goudarzi, ‬‬‬‬‬‬‬‬‬‬ Maryam Farahmandfar

**Affiliations:** 1 Department of Neuroscience and Addiction Studies, School of Advanced Technologies in Medicine, Tehran University of Medical Sciences, Tehran, Iran; 2 Department of Physiology, Faculty of Medicine, Iran University of Medical Sciences, Tehran, Iran; 3 Department of Traditional Medicine, School of Persian Medicine, Tehran University of Medical Sciences, Tehran, Iran; 4 Department of Pharmacognosy, Faculty of Pharmacy, Shahid Beheshti University of Medical Sciences, Tehran, Iran; 5 Department of Pharmacology, Faculty of Pharmacy, Tehran University of Medical Sciences, Tehran, Iran; 6 Electrophysiology Research Center, Neuroscience Institute, Tehran University of Medical Sciences, Tehran, Iran

**Keywords:** Clove essential oil, Eugenol, Intranasal, Seizure, Anticonvulsant, Persian medicine

## Abstract

**Background:**

Epilepsy induces behavioral effects and histological changes in the hippocampus. Eugenol, the main component of clove essential oil, has modulatory effects on seizure. We aimed to investigate the effect of intraperitoneal (IP) and intranasal (IN) clove essential oil on cognitive and histological changes during the chronic phase of temporal lope epilepsy.

**Methods:**

Male Wistar rats were divided into eight groups of seven including control, pilocarpine (PLC), clove oil (IP and IN), sesame oil (IP and IN), phenobarbital (positive control) and saline. Rats were injected with 350 mg/kg PLC to induce status epilepticus (SE). We evaluated the effects of 14 days IP (0.1 ml/kg) and IN (0.02 ml/kg) administration of clove essential oil on locomotor/explorative activity, anxiety-like behavior, spatial recognition memory, and hyperexcitability, as well as hippocampal cell survival in PLC-treated rats.

**Results:**

Our findings indicated that clove oil could effectively ameliorate PLC-induced behavioral deficits, and also alleviate neuronal death in the cornu ammonis 1 (CA1) region of the hippocampus. Behavioral results as in the Y-maze, Open field and elevated plus maze featured significant differences between control and treated groups. Post-seizure behavioral battery (PBSS) results explicated that behavioral hyperexcitability were less in clove oil groups (both IN and IP) compared to PLC-treated rats. Moreover, results of this study demonstrated that IN administration of clove oil was more potent in alleviating behavioral impairment at a lower dosage than by IP route. The results of this study, also demonstrated that intranasal administration of clove oil could reduce duration of recurrent seizures.

**Conclusion:**

In summary, clove oil treatment ameliorated histopathological and behavioral consequences of PLC-induced SE.

## Introduction

Epilepsy is a chronic neurological disorder characterized by spontaneous and recurrent seizures [1]. An imbalance between excitatory and inhibitory neurotransmission has been proposed as the main pathophysiology of epilepsy. Anticonvulsant drugs are used to prevent seizures or reduce their frequency and intensity. Nevertheless, it is estimated that approximately 30–40% of patients continue to suffer from epilepsy despite various medications [2]. Moreover, epilepsy is associated with depression, anxiety, and cognitive dysfunctions like learning disabilities and memory deficits [3]. These comorbidities are caused by several factors including pre-existing brain damage, seizures, or treatments, and have a negative impact on the quality of life in epileptic patients [4]. Owing to the various side effects of anticonvulsants in long-term, high prevalence of comorbidities, and the impact of epilepsy on quality of life, increasing interest is devoted to medicinal plants as a treatment for this condition [5].

Traditional systems of medicine offer a rich source for drug discovery from natural compounds including herbs [6]. Enjoying many millennia of experience and knowledge, Persian Medicine (PM) is a holistic school of medicine, which uses a wide range of *materia medica* to prevent and treat disease [7]. Among the medicinal plants deemed beneficial for several neurologic disorders, including stroke, epilepsy, amnestic disorders, and specific types of headaches is clove. According to PM literature, this herb is also a brain tonifier, strengthens the mind and reinforces cogitation [8, 9]. In addition to being prescribed as single therapy or in drug formulas peroral, other methods of drug administration including intranasal route are advised by Persian scholars to achieve stronger effects, especially tonifying and strengthening effects on the brain [10].

*Eugenia caryophyllata*, commonly known as clove, is a plant from the Myrtaceae family. The essential oil isolated from the buds or leaves is widely used for medicinal properties. Clove essential oil contains several bioactive compounds such as eugenyl acetate, eugenol, β caryophyllene, and oleanolic acid [11]. Eugenol is the major component of clove oil present in concentrations of 80–90% in clove bud oil, and 82–88% in clove leaf oil [12]. It has broad biological protective activities against nephrotoxicity, chronic inflammation, and cancer [13–15]. Being a hydrophobic molecule, eugenol is able to cross the blood–brain barrier, and thus, seems to be effective in treating a variety of neurological disorders [16]. Antioxidative, immunomodulatory, and antiapoptotic properties, make this compound a potentially efficient neuroprotective [17]. Prior findings suggest that eugenol is protective against neurotoxicity induced by oxidative stresses [17–20].

A number of studies have investigated the anticonvulsant activity of eugenol and demonstrated its ability to reduce epileptic-like activity in cortical slices [21]. In the hippocampus, eugenol decreases severity and duration of seizures, as well as mortality via reducing mossy fiber sprouting and increasing glutathione peroxidase (GPx) antioxidant [22, 23]. It can also inhibit granule cell dispersion (GCD) which is frequently seen in the hippocampus of epileptic patients, and thereby prevent the epileptogenic process [24].

Furthermore, clove essential oil has anti-stress and antidepressant properties [25]. The main component of this oil, eugenol, can enhance locomotor activity [26], and can thus ameliorate depressive-like behaviors. Clove oil has also been shown to improve memory and reverse learning and memory deficits [27–29]. Considering antiepileptic, neuroprotective, and cognitive effects, clove may be a good drug target for epilepsy and related morbidities. To our knowledge, not enough research has been conducted on effects of clove oil on behavioral changes in pilocarpine (PLC) model of epilepsy.

In previous studies on seizures, drugs have been administered via oral, or intraperitoneal (IP) routes [30, 31]. Intranasal (IN) drug administration is one of the therapeutic methods that has been considered by scientists in the recent years [32]. The nasal cavity has a dense vascular network that provides a direct route for drugs to pass through the mucous membranes into the brain bloodstream. Moreover, intranasal administration bypasses gastrointestinal destruction and primary hepatic metabolism. Hence, this drug delivery route effectively increases drug bioavailability, and enables faster onset of action [33, 34].

Given the above, the aim of this study was to evaluate the effect of intranasal and intraperitoneal administration of clove oil on spatial memory, anxiety, locomotor activity and epileptic seizures in the chronic phase caused by chemical kindling in a rat model of epilepsy.

## Methods

### Animals

The study was performed on adult Sprague Dawley male rats weighing 220–250 g. Animals were housed under standard conditions with 22 ± 2 °C temperature, 12-h light/dark cycle, 45 ± 5%humidity, and free access to food and water. All animal experiments were carried out in accordance with the guidelines of National Institute of Health for the Care and Use of Laboratory Animals (NIH Publication No. 80–23; revised 1978) and were approved by the Ethics Committee of Tehran University of Medical Sciences (IR.TUMS.VCR.REC.1395.283).

### Experimental design

Fifty-six rats were randomly divided into eight groups (seven rats in each group) comprising: 1. Control (no manipulation); 2. Pilocarpine (PLC) group (370 mg/kg, IP); 3. PLC + clove oil (0.1 ml/kg, IP, 14 days), 4. PLC + clove oil (0.02 ml/kg, IN, 14 days), 5. PLC + Sesame oil (1 mL, IP, 14 days), 6. PLC + Sesame oil (25 µL in each nostril, IN, 14 days), 7. PLC + Phenobarbital (40 mg/kg, IP, 14 days), 8. PLC + Saline (1 ml, IP, 14 days). Throughout the experimental procedures, animals that were lost, were replaced by another.

Seizures were induced by IP injection of pilocarpine (370 mg/kg, Sigma Aldrich). In order to limit peripheral muscarinic effects of pilocarpine, methylscopolamine bromide (1 mg/kg) was administered 30 min before pilocarpine injection. Subsequently, rats were monitored for 3–4 h and seizure severity was graded according to Racine’s scale [35].

Accordingly, seizures were scored in each rat with modified Racine’s scale based on the behavioral assessment of only convulsive (motor) seizures. Seizure stages were as follows: 0. normal behavior; 1. vibrissae twitching and facial automatisms; 2. facial and head clonus; 3. unilateral forelimb clonus; 4. rearing with bilateral forelimb clonus; and 5. loss of balance and falling accompanied by generalized clonic (GC) convulsions. Latency was defined as the average time duration (Sec) between pilocarpine injection and first appearance of convulsive behavior.

Only rats in stages four and five of seizure scoring were included in this study. Also, diazepam (2.5 mg/kg) was administered 3 to 4 h after the onset of seizures to limit the duration of status epilepticus (SE) in epileptic rats. Subsequently, animals were fed Hartmann’s solution in the recovery duration [36]. Two weeks after pilocarpine administration, and during chronic phase, the occurrence of spontaneous seizures were recorded by 8 h of monitoring per day for two weeks.

### Essential oil preparation

Dried buds and leaves of *Eugenia caryophyllata* were obtained from a local herbal market in Tehran and authenticated by M. Kamalinejad at the Department of Pharmacognosy, Shahid Beheshti University of Medical Sciences, Tehran, Iran. The voucher specimen coded E-542 has been deposited at the herbarium number of Faculty of Pharmacy of Shahid Beheshti University of Medical Sciences. Essential oil extraction was performed at the Botanical Research Center of Shahid Beheshti University of Medical Sciences. Dried leaves of *Eugenia caryophyllata* were distilled for 4 h using a Clevenger apparatus, with 5% yield. The obtained clove essential oil (with a specific gravity of 1.04 g/ml) was added to sesame oil and shaken vigorously to prepare 0.1 ml/ml concentrations before administration to animals. Steam distillation as a co-distillation technique was accomplished to separate components of this mixture by using live steam.

### Clove oil treatment

Clove oil was administered at 0.05 and 0.1 doses, but there were not any meaningful differences between results. According to our study goals, we decided not to point this out. Besides, 0.1 ml/kg clove oil was more effective according to results of dose–response analysis. Studies that are referenced in manuscript have indicated that 0.1 is the most effective dose.

In the chronic phase of epilepsy, rats were post-treated by clove oil or its solvent (sesame oil) Intraperitoneally (IP, 0.1 ml/kg) or intranasally (IN, 0.02 ml/kg) for 14 days. The total volume of IP injection was approximately 0.25 mL for each rat. Regarding the intranasal route, drugs were delivered into the nose using a 25 µL micropipette and gel loading pipette tip, while the rat was held upside down. To minimize drug loss and allow maximum drug volume to run down the nasal cavity, drugs were infused slowly over approximately 20 s [37]. The total volume administered intranasally was 50 µl (25 µL in each nostril).

All drugs were administered between 8 a.m. and 10 p.m. Rats were transferred to their cage after treatment.

### Behavioral assessment

Different behavioral tests were used to investigate excitability, anxiety, and spatial memory of treated rats with pilocarpine and clove essential oil. Behavioral tests including open field, Y-maze, elevated plus maze and hyperexcitability tests were performed 14 days after drug administrations. All behavioral tests were performed during one week, from 10 a.m. until 1 p.m. and each group had only one test in each day. Time duration of each test was specific according to test protocol.

#### Open field test

The effects of treatments on spontaneous locomotor/explorative activity and anxiety-like behavior were assessed by open field test [38]. The open field was an acrylic, 60 × 60 × 40 cm square box with a center zone in the middle marked with a permanent marker (25 × 25 cm). Animals were placed individually in the center of the arena and allowed to explore the open field. Animal behavior was recorded by a video camera (eye vision, v 2.3.5) mounted above the open field, and total distance traveled and time spent in the center of the arena were analyzed using a tracking system (EthoVision, Noldus, The Netherlands). Before each trial, the field was cleaned thoroughly with 70% ethanol solution.

#### Y-maze

Spatial recognition memory was assessed by recording spontaneous alternation behavior in a single session Y-maze [39]. The Y-maze apparatus consisted of three equal arms with the dimensions of 30 × 20 × 10 cm labeled as A, B and C. The arms converged in an equilateral triangular central area that was 15 cm at its longest axis. Each rat was placed at the end of one arm and allowed to move freely through the maze during an 8-min session. The series of arm entries was recorded visually. Arm entry was considered to be completed when the base of the animal’s tail had been completely placed in the arm. Alternation was defined as successive entries into the three arms on overlapping triplet sets. The maximum number of possible spontaneous alternations was determined as the total number of arms entered—2, and the percentage was calculated as the ratio of actual to possible alternations × 100.

#### Elevated plus maze

Elevated plus maze (EPM) is a validated apparatus for measuring anxiety-like behavior in rodents. This apparatus consists of four elevated arms above the floor, arranged in two opposite closed arms, two opposite open arms, and a central platform. Each animal was placed in the central zone of the EPM and behavior was recorded for 5 min using a video camera. To measure anxiety-related behavior, the number of open arm entries and the percentage of time spent in the open arms (open arm/open + enclosed arms × 100) were calculated.

#### Hyperexcitability tests

Post-seizure behavioral battery (PSBB) tests described by Rice et al. are used to assess hyperexcitability differences between epileptic and control rats [40]. This battery comprises four tests including:

1. Approach response test: A pen held vertically is moved slowly toward the animal's face. Responses are scored as 1. no reaction; 2. The rat smells the pen, 3. the rat moves away from the pen; 4: the rat freezes; 5, the rat jumps away from the pen; and 6: the rat jumps at or attacks the pen.
2. Touch response test: The animal is gently prodded in the rump with the blunt end of a pen. Responses are scored as 1. no reaction; 2. the rat turns toward the pen; 3. the rat moves away from the pen; 4. the rat freezes; 5. The rat returns to the touchpoint; 6. the rat turns away from the touch; and 7. the rat jumps with or without making a sound.
3. Finger-snap test: A hand click is made several centimeters above the rat's head. Responses are scored as 1. no reaction; 2. the rat jumps slightly or flicks the ear (normal reaction); 3. the rat jumps drastically.
4. Pick-up test: The rat is picked up by grasping around the body. Responses are scored as 1. very easy, 2. easy with vocalizations, 3. some difficulty, the rat rears and faces the hand, 4. difficult, the rat avoids the hand by moving away, 5. very difficult, the rat attacks the hand.

The test battery was carried out repeatedly in each animal at different time points (days 2, 4, 6, 8, 10, 12 and 14) after treatment. There were three observers for each rat, and the means of their scores were calculated for each rat per test. Tests were performed in animal cages with a 30-min interval between tests.

#### Monitoring of behavioral seizure

Frequency and duration of spontaneous recurrent seizures (SRS), were monitored 20 h a day for 5 days following treatment.

### Nissl staining

Histological analysis was performed by Nissl staining. Animals were deeply anesthetized with ketamine (100 mg/kg) and were perfused transcardially with saline followed by 4% paraformaldehyde. Brains were removed and post-fixated 48 h in paraformaldehyde, and then processed, and embedded in paraffin. Coronal paraffin sections of the hippocampus (8 µm thick) at the level of – 3.80 mm to – 4.1 mm bregma [41], were stained according to the Nissl method [42]. Briefly, tissue sections were deparaffinized in xylene, rehydrated by descending graded ethanol (100, 95, 70%), stained in fresh 1% cresyl violet, decolorized in acetic acid, dehydrated through ascending graded ethanol (70, 95, 100%), cleared in xylene and cover slipped using DPX mounting medium. Stained sections were observed under an optical microscope (Olympus CK2; Olympus Optical Co., Japan). In three consecutive slices of the hippocampal cornu ammonis 1 and 3 (CA1 and CA3) subregions, the pyramidal cells were counted by ImageJ software (National Institute of Health, USA) at a final magnification of 400 x.

### Data analysis

Statistical analyses were performed with GraphPad Prism 8.0. All data are expressed as mean ± SEM. The normality of data distribution was assessed by the Kolmogorov–Smirnov test, which indicated that the data were normal. To compare the experimental groups, one-way analysis of variance (ANOVA) was used and followed by Tukey's Test for Post-hoc Analysis. Data from hyperexcitability tests were analyzed using two-way ANOVA with repeated measures. Statistical significance was defined as a *p*-value of < 0.05.

## Results

### Results of behavioral assessment

#### Open field test

**Fig. 1:**
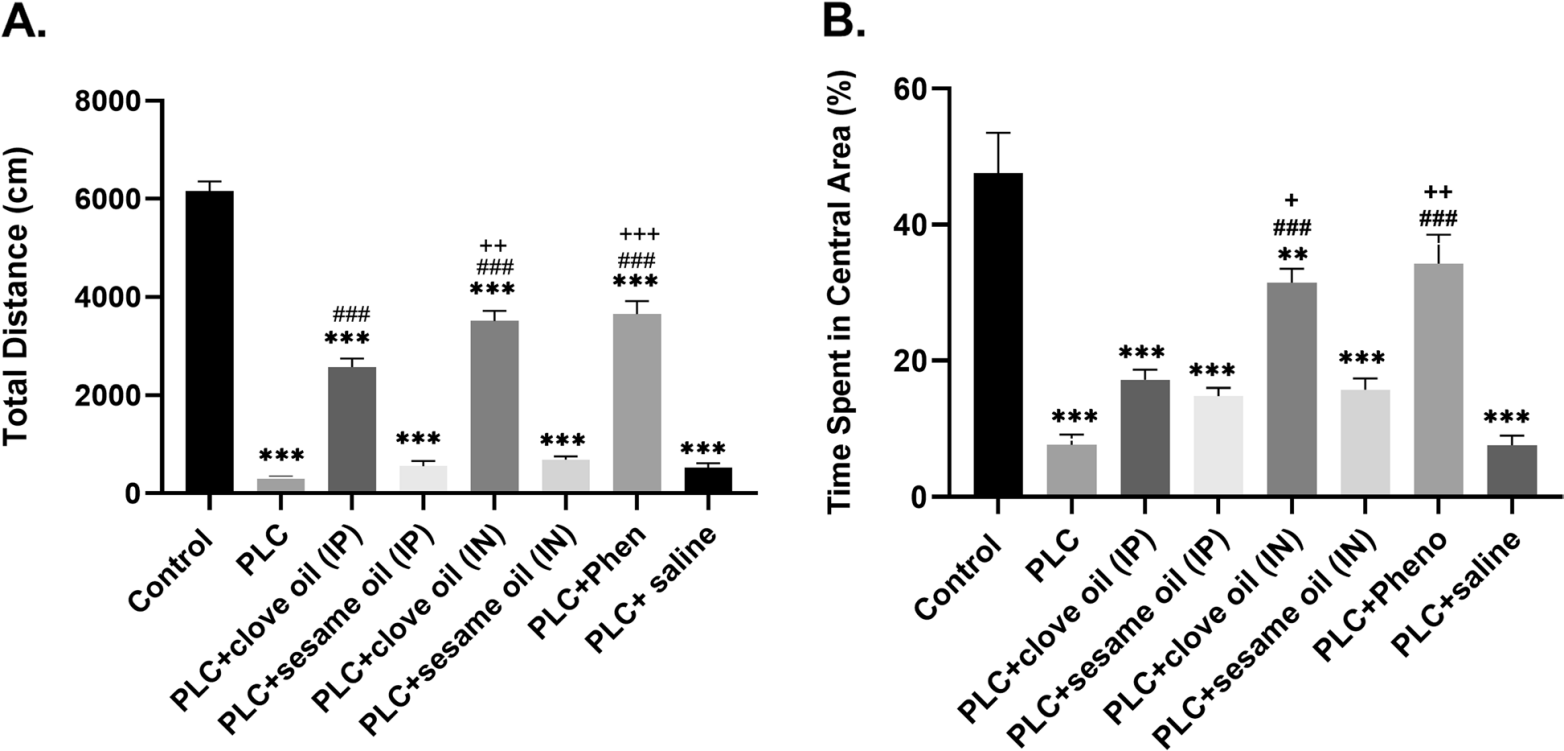
Results of activity/exploration (**A**) and anxiety-like behavior (**B**) in open field test. Data are shown as mean ± SEM. *** *P* < 0.001 (vs. Control), ### *P* < 0.001 (vs. PLC), + *P* < 0.05 (vs. PLC + clove oil (IP) group), +  + *P* < 0.01 (vs. PLC + clove oil (IP) group), +  +  + *P* < 0.001 (vs. PLC + clove oil (IP) group)

The open-field test was carried out to assess activity/exploration and anxiety-like behavior in experimental groups. One-way ANOVA analysis showed significant difference in total distance traveled between groups (df = (7,48), f = 147.6, *p* < 0.001, *n* = 7 in each group). As shown in Fig. 1, our results indicated a significant difference in locomotor activity/exploration between PLC and the control group (321.5 ± 22.57 vs 6155 ± 200.2, *P* < 0.001). Locomotor activity/ exploration improved after treatment with clove oil through both IP and IN delivery routes (2568 ± 175.8 and 3509 ± 210.5 vs 321.5 ± 22.57, *P* < 0.001), and also in the rats treated with phenobarbital compared with the PLC group (3652 ± 263.8 vs 321.5 ± 22.57, *P* < 0.001). Locomotor activity/exploration improvement was higher in rats that received clove oil by IN route (3509 ± 210.5 vs 2568 ± 175.8, *P* < 0.01), and rats that received phenobarbital (3652 ± 263.8 vs 2568 ± 175.8, *p* < 0.001) compared with the rats that received clove oil by IP route (Fig. 1A).

Moreover, One-way ANOVA analysis showed significant difference in anxiety-like behavior between groups (df = (7,36), f = 23.54, *p* < 0.001, *n* = 7 in each group). Multiple comparisons indicated that the total time spent in the central region of the open field in the PLC group was significantly less than the control group (7.67 ± 1.48 vs 47.5 ± 5.93, *P* < 0.001). As shown in Fig. 1B, treatment with clove oil via IN route (31.45 ± 2.05 vs 7.67 ± 1.48, *P* < 0.001), and also with phenobarbital (34.29 ± 4.2 vs 7.67 ± 1.48, *P* < 0.001) could effectively alleviate anxiety-like behavior in comparison with the PLC group, as well as the rats treated by clove oil via IP route (31.45 ± 2.05 vs 17.17 ± 1.51, *p* < 0.05 & 34.29 ± 4.2 vs 17.17 ± 1.51, *p* < 0.01 respectively). This was indicated by more time spent in the center of the open field by animals of these groups.

#### Y-maze

**Fig. 2:**
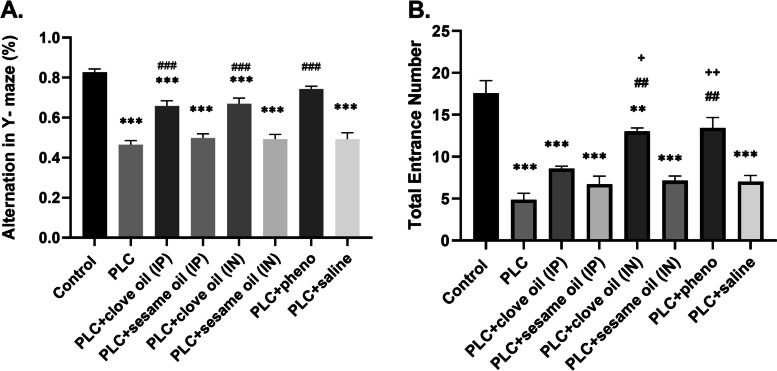
Alternation behavior (**A**) and total entrance frequency (**B**) in the Y-maze task. Values are presented as means ± SEM. ** *p* < 0.01 (vs. Control), *** *p* < 0.001 (vs. Control), ## *p* < 0.01 (vs. PLC), ### *p* < 0.001 (vs. PLC), + *p* < 0.05 (vs. PLC + clove oil (IP) group), +  + *p* < 0.01 (vs. PLC + clove oil (IP) group)

Short-term spatial recognition memory was evaluated by alternation behavior in the Y-maze task. One-way ANOVA analysis indicated a significant difference between groups (df = (7,48), f = 34.45, *p* < 0.001, *n* = 7 in each group). In the comparison between the groups, the alternation percentage of the PLC-injected rats was found to be considerably lower than that of the control group (0.46 ± 0.02 vs 0.82 ± 0.01, *p* < 0.001). Clove oil-treated (both IP and IN) (0.49 ± 0.02 and 0.67 ± 0.02 vs 0.46 ± 0.02, *p* < 0.001), and also phenobarbital-treated rats showed a significant increase in alternation percentage as compared to PLC group (0.74 ± 0.01 vs 0.46 ± 0.02 p < 0.001), which indicates improvement of spatial memory in these groups; However, this memory improvement did not reach the level of the control group (0.49 ± 0.02 and 0.67 ± 0.02 and 0.74 ± 0.01 vs 0.82 ± 0.01, *p* < 0.001). No significant difference was observed in alteration percentage between groups that received clove oil intraperitoneally and intranasally (Fig. 2A).

One-way ANOVA analysis of the total number of arms entered, as an index for locomotor activity, showed a significant difference between groups (df = (7,48), f = 23.65, *p* < 0.001, *n* = 7 in each group). Tukey's analysis showed considerable difference between the PLC injected rats and the control group (4.8 ± 0.7 vs 17.57 ± 1.49, *p* < 0.001p). Administration of clove oil through IN route caused substantial enhancement in the total number of entered arms compared to the PLC group (13.00 ± 0.43 vs 4.8 ± 0.7 *p* < 0.01). Such improvement in locomotor activity was also observed in the phenobarbital group in comparison with the PLC group (13.43 ± 1.25 vs 4.8 ± 0.7 *p* < 0.001). Meanwhile, a significant increase was observed in the total number of arm entries in Y-maze in the PLC + clove oil (IN) (13.00 ± 0.43 vs 6.7 ± 0.9 *p* < 0.05) and PLC + phenobarbital groups compared with PLC + clove oil (IP) ((13.43 ± 1.25 vs 6.7 ± 0.9 *p* < 0.01), (Fig. 2B).

#### Elevated plus maze

**Fig. 3:**
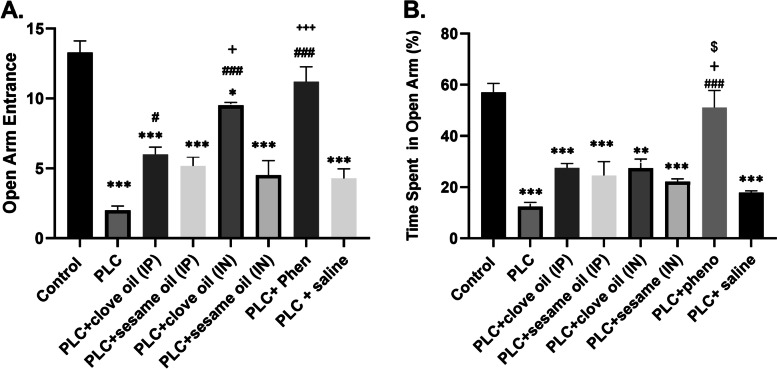
Evaluation of anxiety-like behaviors in experimental groups. Results of the time spent (**A**) and percentage of entries (**B**) in the open arms of the EPM. Values are presented as means ± SEM. * *P* < 0.05 (vs. Control), ** *P* < 0.01 (vs. Control), *** *P* < 0.001 (vs. Control), ## *p* < 0.01 (vs. PLC), ### *p* < 0.001 (vs. PLC), + *p* < 0.05 (vs. PLC + clove oil (IP) group), $ *p* < 0.05 (vs. PLC + clove oil (IN) group)

In EPM, the number of entries and percentage of time spent in the open arms was considered an anxiety-like behavior. Analysis of the number of open arm entries demonstrated significant difference between groups (df = (7,42), f = 31.57, *p* < 0.001, *n* = 7 in each group). Based on post hoc analysis, there was a remarkable difference in the number of open arms entries in the PLC-injected group compared with the control group (2.0 ± 0.3 vs 13.29 ± 0.83, *P* < 0.001). Our findings revealed that the animals treated with clove oil, both IN and IP, show lower anxiety-like behavior as they have significant further open arm entries compared with the PLC group (9.5 ± 0.2 and 6.0 ± 0.5 vs 2.0 ± 0.3, *P* < 0.001 & *P* < 0.05 respectively; Fig. 3A). Also, this betterment was observed in PLC + phenobarbital group compared with the PLC group (11.20 ± 1.06 vs 2.0 ± 0.3, *P* < 0.001). Between the two routes of clove oil delivery, IN was more effective than IP administration (9.5 ± 0.2 vs 6.0 ± 0.5, *P* < 0.05). Delivery of clove oil through IN route was as effective as phenobarbital with no difference observed between them (Fig. 3A).

Also, one-way ANOVA analysis for percentage of time spent on open arm showed significant difference between groups (df = (7,32), f = 14.25, *p* < 0.001, *n* = 7 in each group). PLC rats spent a lower percentage of time in the open arms compared with the control group (12.33 ± 1.67 vs 57.03 ± 3.45, *P* < 0.001). Clove oil treatment did not change the percentage of time spent in the open arm. In contrast, phenobarbital was able to effectively increase this time in comparison with PLC group (51.07 ± 6.7 vs 12.33 ± 1.67, *P* < 0.001), and also in comparison with IP (51.07 ± 6.7 vs 27.47 ± 1.76, *P* < 0.05), and IN (51.07 ± 6.7 vs 27.44 ± 3.5, *P* < 0.05, *P* < 0.05) treated groups (Fig. 3B).

#### Behavioral hyperexcitability test

**Fig. 4:**
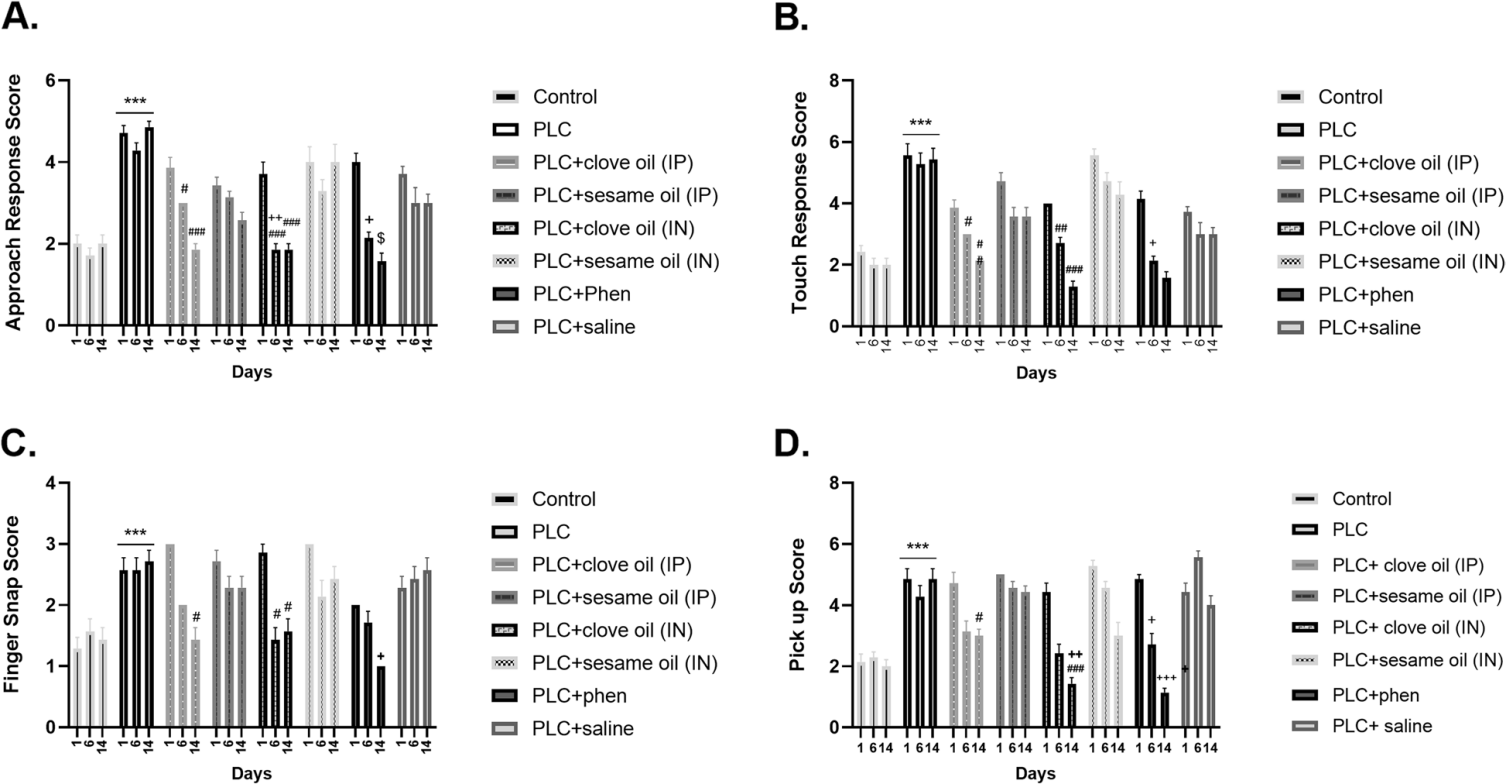
Evaluation of post-seizure behavioral changes including Approach-Response (**A**), Touch-Response (**B**), Pick-up (**C**), and Finger-Snap (**D**) tests. Values are presented as means ± SEM. *** *P* < 0.001 (vs. Control), # *p* < 0.05 (vs. PLC), ## *p* < 0.01 (vs. PLC), ### *p* < 0.001 (vs. PLC), + *p* < 0.05 (vs. PLC + clove oil (IP) group), +  + *p* < 0.01 (vs. PLC + clove oil (IP) group), +  +  + *p* < 0.001 (vs. PLC + clove oil (IP) group), $ *p* < 0.05 (vs. PLC + clove oil (IN) group)

Behavioral hyperexcitability was assessed by two-way ANOVA with repeated measure analysis of PSBB tests including approach response test (df = (14, 96), f = 5.58, *p* < 0.001), touch response test (df = (14, 96), f = 4.5, *p* < 0.001), finger snap test (df = (14, 96), f = 5.57, *p* < 0.001) and pick-up test (df = (14, 96), f = 13.45, *p* < 0.001). The results of each test are illustrated separately in Fig. 4 (Fig. 4 A to D). In all tests, PLC treated epileptic rats are more easily agitated compared to control animals, indicating that epileptic rats are more sensitive to environmental stimuli (*P* < 0.001). Between-group comparisons suggested that both routes of drug administration (IP and IN) could reduce hyperexcitability in treated groups compared with PLC rats. This improvement started from day 6 and continued until day 14.

In the approach response test, on the 6^th^ day of treatment, clove oil through IN route was more effective in ameliorating hyperexcitability behaviors than IP route (*P* < 0.01). In touch response and also finger snap test, no remarkable difference was observed in either 6^th^ or 14^th^ post-treatment days between IP and IN delivery routes. In pick-up test, rats who received clove oil by IN route indicated less excitability than the IP-treated group (*P* < 0.01).

#### Hippocampal cell count

**Fig. 5:**
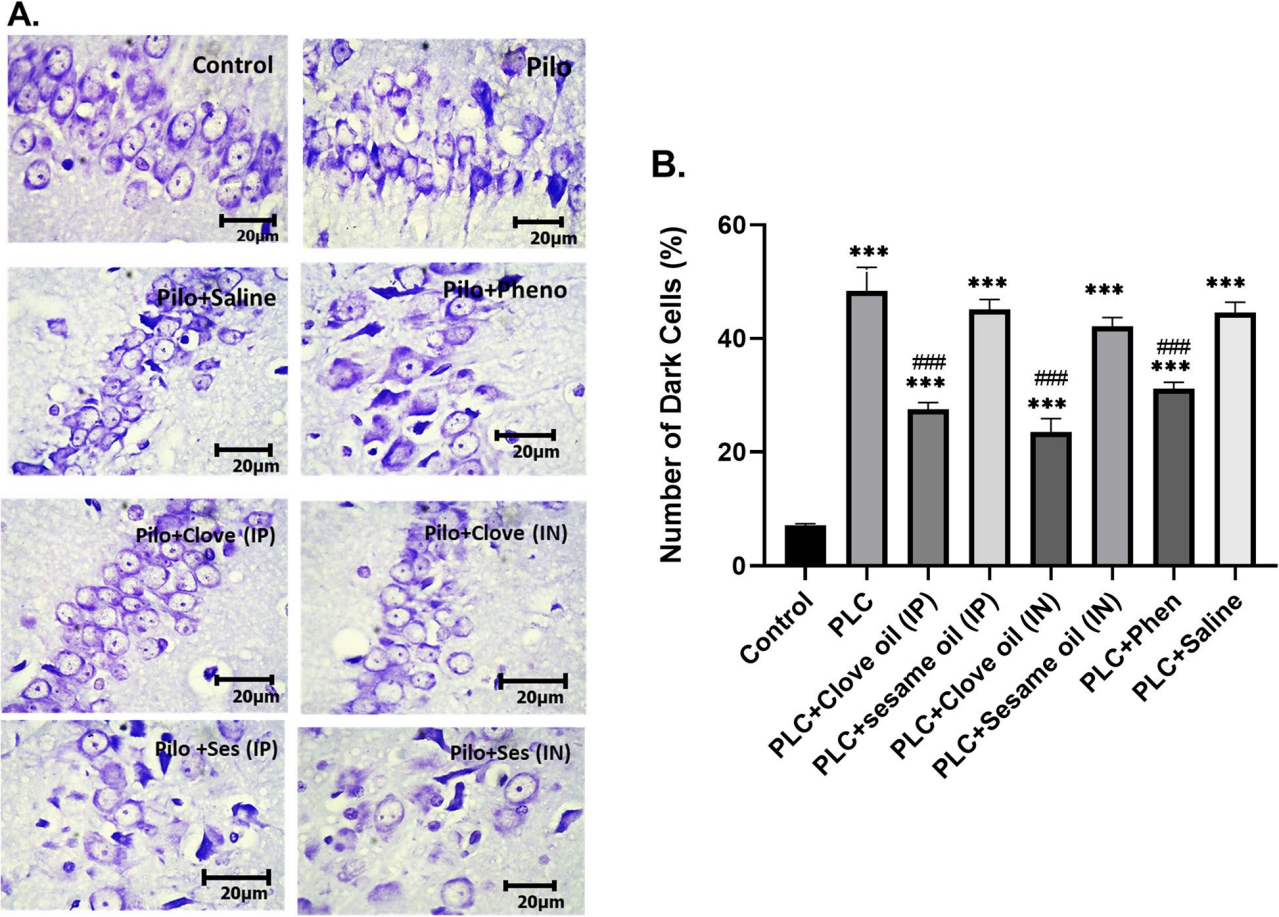
Results of Nissl‐stained cell number in the hippocampal CA1 region. Photomicrographs of the hippocampus CA1 region cells in 400 × magnification (**A**) and a bar graph showing quantitative results of damaged neurons (dark cells) in the CA1 region (**B**). Values are presented as means ± SEM. *** *P* < 0.001 (vs. Control) and ### *P* < 0.001 (vs PLC)

Histological analysis performed by Nissl staining. One-way ANOVA analysis showed significant difference between groups (df = (7,16), f = 46.8, *p* < 0.001, *n* = 3 in each group). As shown in Fig. 5, results of Nissl staining displayed that the numbers of damaged neurons in the hippocampal CA1 region were increased dramatically in the PLC group compared with the control group (48.4 ± 4.1 vs 7.1 ± 0.32, *p* < 0.001). Loss of hippocampal neurons was attenuated after treatment by clove oil via both IP and IN delivery routes in the PLC + clove oil groups (23.53 ± 2.3 & 27.56 ± 1.24 respectively vs 48.4 ± 4.1, *P* < 0.001), and also after treatment by phenobarbital (31.17 ± 1.11 vs 48.4 ± 4.1; *p* < 0.001) compared with the PLC group. Quantitative analysis revealed that clove oil administration through both IP and IN routes could reduce CA1 neuronal loss as much as phenobarbital. However, in these PLC + treatment groups, the number of CA1 dark cells was still significantly higher than the control group (23.53 ± 2.3 & 27.56 ± 1.24 vs 7.1 ± 0.32, *P* < 0.001). No significant difference was observed between PLC + IP and PLC + IN groups (Fig. 5).

#### Duration and frequency of SRS

**Fig. 6:**
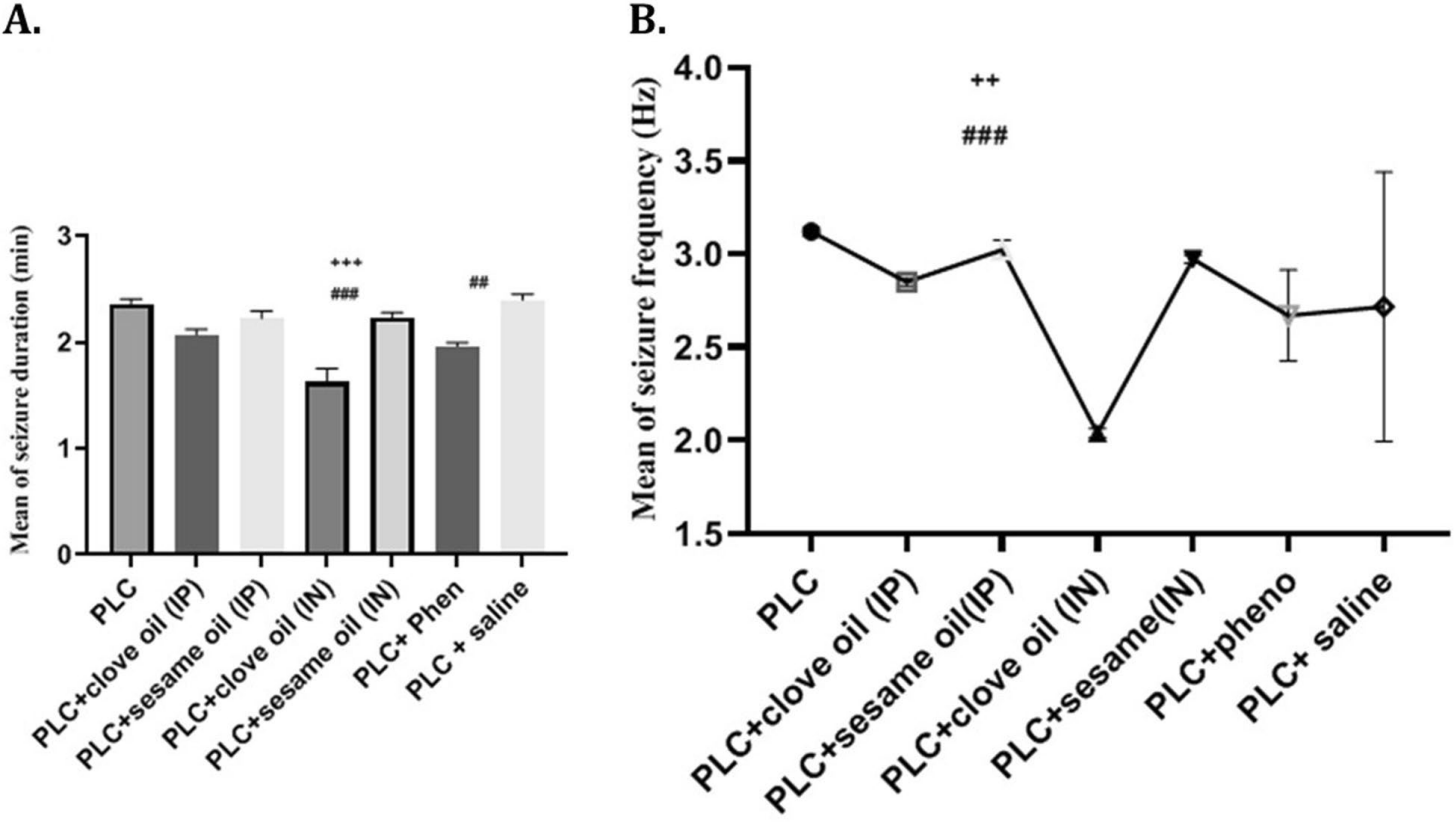
Results of video monitoring of groups regarding changes in duration (**A**) and frequency (**B**) of spontaneous recurrent seizures. Values are presented as means ± SEM. ## *p* < 0.01 (vs. PLC), ### *p* < 0.001 (vs. PLC), + *p* < 0.05 (vs. PLC + clove oil (IP) group)

After two weeks of treatment, rats were monitored, and the average frequency and duration of SRS was investigated following 14 days of drug administration. The results showed that intranasal administration of clove oil markedly reduces the frequency and duration of the recurrent seizures (Fig. 6). One-way ANOVA analysis indicated a significant difference between groups (df = (6,42), f = 15.94, *p* < 0.001, *n* = 7 in each group).

## Discussion

In this study, we evaluated the effects of clove oil administration on histological and behavioral improvement in the PLC-induced SE rat model, and also compared the efficacy of intranasal (IN) and intraperitoneal (IP) administration routes.

One of the most common models of temporal lobe epilepsy (TLE) in rodents is the PLC model. Systemic administration of this cholinergic muscarinic agonist leads to evocation of SE in rats and mice, with manifestations of spontaneous recurrent seizures, and hippocampal destruction that is similar to hippocampal sclerosis in TLE patients [43, 44].

Our finding showed that PLC treatment leads to spontaneous seizure followed by hippocampal neuronal loss and adverse behavior symptoms including impairment in locomotor/exploring activity and spatial memory, as well as hyperexcitability, and anxiety-like behavior, all in accordance with previous studies [45]. A perturbation in glutamatergic (excitatory)/ gamma-Aminobutyric acid (GABA)-ergic (inhibitory) balance has been implicated in the pathophysiology of epilepsy [46]. Evidence supports the role of glutamate receptors in initiating and increasing seizure activity [47]. PLC-induced seizures are mediated by continuous increase in extracellular hippocampal glutamate levels after PLC perfusion [48]. Moreover, a significant deficit of GABAergic function was shown in the PLC model of SRS [49].

After two weeks of treatment, the most effective reduction in both average frequency and duration of SRS was observed in the IN clove oil group. Previous studies have shown anticonvulsant properties for *Eugenia caryophyllata* [50, 51]. Clove oil prevents maximal electroshock (MES)-induced tonic seizures, and nearly doubles the threshold of clonic pentylenetetrazole (PTZ)-induced seizures [52]. It has also been demonstrated that both aqueous and ethanol extracts of clove increase latency to the first minimal clonic seizure and generalized tonic–clonic seizure in mice treated with PTZ [53]. Clove essential oil is comprised of several bioactive components such as eugenol, β-caryophyllene, eugenyl acetate, α-humulene and carvacrol [54]. The main compound of clove oil, eugenol, is reported to modulate brain functions by regulating Na^+^ and Ca^2+^ voltage-gated channels and releasing neuroransmitters [55]. A reversible, dose-dependent inhibition of GABA-induce currents has also been demonstrated for this compound [56]. Eugenol affects the firing of neuronal action potential and modulates neuronal hyperexcitability in PLC-induced temporal lobe seizures [57]. Other studies have demonstrated antiepileptic activity of this compound in PTZ-treated mice [58, 59]. It should be noted, however, that at higher doses, this compound has been shown to reversibly increase the frequency of action potentials, and thereby induce epileptiform activity [60].

Antiepileptic and neuroprotective properties of other clove oil compounds have also been observed in studies. Carvacrol ameliorates convulsant activity in rodents, probably via the GABAergic neurotransmitter system [61, 62]. It can also prevent recurrent seizures, cell death, and cognitive decline following SE [63]. There is evidence that humulene has some binding affinity to GABA–benzodiazepine receptor, via which an anticonvulsant effect is exerted [64, 65]. GABA–benzodiazepine receptor agonism, and inhibition of nitric oxide synthesis have been proposed as mechanisms for the antiepileptic activity of β-caryophyllene [66]. Acting as a cannabinoid receptor 2 (CB2) agonist, this compound can protect mice against MES-induced seizures, and also ameliorate cognitive impairments and neurotoxicity [67]. It also exerts neuroprotective effects against focal cerebral ischemia–reperfusion injury and dopaminergic neuron injury [68–70].

One of the aims of this study was to explore the behavioral effects of clove oil on PLC-induced epilepsy. This was investigated interms of locomotor/explorative activity (open field test), anxiety-like behavior (EPM), spatial recognition memory (y-maze), and hyperexcitability (PSBB). Based on results of the open field test, locomotor activity/exploration was enhanced after treatment with phenobarbital and also clove oil. Clove oil by IN route was more effective than IP route, and almost as effective as phenobarbital. Assessing anxiety behavior via EPM, was also indicative of lower anxiety-like behavior in animals treated with clove oil (both IN and IP) and phenobarbital groups, again with higher efficacy of the IN route. However, clove oil treatment did not change the percentage of time spent in the open arm. Regarding behavioral hyperexcitability, PSBB test results showed that clove oil groups (both IN and IP) were less agitated and less sensitive to environmental stimuli compared to PLC-treated rats. Alternation behavior in the Y-maze task demonstrated significant increase in both IP and IN clove oil groups, indicative of short-term spatial recognition memory improvement.

Our results were consistent with previous studies on the effects of clove oil and its major constituent, eugenol on behavior and memory. Attributed to inhibition of monoamine oxidase, clove oil reduces anxiety [71]. It has also been demonstrated to improve locomotor acitivity and decrease depression [72]. Likewise, eugenol has shown anxiolytic activities in a number of studies [73]. It can down-regulate expression of neurokinin 1 receptor protein, a G protein coupled receptor, involved in affective behaviors [74].

Regarding memory, clove oil has significant effects on amelioration of scopolamine-induced retention memory deficit [75]. This effect includes both short-term and long-term memory, and is to some extent ascribed to its anti-oxidant property [76]. Similarly, eugenol can enhance spatial and recognition memory [31] and increase learning-memory abilities [77]. In a recent study exploring these effects, a descrease in spontaneous alteration behavior was seen in Y-maze test [78]. Moreover, pretreatment of rats with eugenol has been shown to enhance locomotor functions and short-term memory in rats with traumatic brain injury [79].

Based on histological findings of this study, loss of hippocampal neurons was attenuated by clove oil. Although the number of CA1 dark cells was still significantly higher than the control group, both IP and IN administration of clove oil reduced CA1 neuronal loss as effectively as phenobarbital. These results were also in line with previous research. Neuroprotective effects of eugenol have partly been attributed to down-regulation of NF-κB and MAPK signaling pathways [80]. A recent study has demonstrated that eugenol increases neurogenesis, and dendritic complexity of neurons in the dentate gyrus and CA1 basal region in mice [31]. Also, pretreatment of rats with eugenol lowers neuronal cell loss in traumatic brain injury [79].

One of the main goals of our study was to study effects of IN administration of clove oil in comparison with IP route. Our results indicated that IN administration of clove oil was more potent than IP route at a lower dosage in a number of tests including the open-field test, total entrance frequency in the Y-maze task, and the number of entries in the open arms in EPM. Moreover, Clove oil by IN route was more effective than phenobarbital or clove oil by IP route in decreasing frequency and duration of seizures.

Being a noninvasive route that possesses benefits such as rapid absorption, avoidance of the first-pass metabolism, and convenience in use and self-medication, intranasal administration has been taken into consideration for treatment of neurological diseases during the past decades [81]. Optimal nasal mucosa conditions, including rich blood flow and the close anatomical relevance to the brain, ensure adequate and fast absorption and diffusion of drugs [82]. Previous studies have shown that IN administration of benzodiazepines is very useful in epilepsy, when rapid cessation of seizure activity is crucial [32]. A bioavailability of 50–83% has been reported for intranasally-administered midazolam compared with intravenous route [32]. The behavioral and histological effects of IN administration of clove oil in epilepsy have not previously been investigated to our knowledge, however, there are reports of modulatory effects of clove essential oil aroma on pain intensity and anxiety [83].

## Conclusion

Our data revealed that 14 days of treatment with clove oil could effectively alleviate PLC-induced histological and behavioral deficits in epileptic rats. Moreover, administration of clove oil by IN route was the most effective treatment in reduction of frequency and duration of PLC-induced seizures. Based on study results, intranasally-administered clove oil showed an overall higher efficacy at a lower dosage compred with intraperitoneal route. Clove oil may mediate its behavioral effects through the regulation of many molecular targets such as antioxidant [84, 85] and anti-apoptotic [84] factors or modulation of neurotrophic factors [86] and gene expression of GABA and NMDA receptors [56] in the brain. However, to clarify the exact mechanism(s) involved, more studies are required. Future studies, including clinical trials are suggested to explore this potential drug in management of epilepsy and its comorbidities.

## Data Availability

The datasets used and/or analysed during the current study are not publicly available due to Tehran University of Medical Sciences policies, but can be provided by the corresponding author on reasonable request.

## Abbreviations

CA1: And cornu ammonis 1
CA3: Cornu ammonis 3
CB2: Cannabinoid receptor 2
EPM: Elevated plus maze
GABA: Gamma-aminobutyric acid
GCD: Granule cell dispersion
GPx: Glutathione peroxidase
IN: Intranasal
IP: Intraperitoneal
MES: Maximal electroshock
PLC: Pilocarpine
PM: Persian medicine
PSBB: Post-seizure behavioral battery
PTZ: Pentylenetetrazole
SE: Status epilepticus
SRS: Spontaneous recurrent seizures
TLE: Temporal lobe epilepsy

## References

[1] Stafstrom CE, Carmant L (2015). Seizures and epilepsy: an overview for neuroscientists. Cold Spring Harb Perspect Med.

[2] Laxer KD, Trinka E, Hirsch LJ, Cendes F, Langfitt J, Delanty N (2014). The consequences of refractory epilepsy and its treatment. Epilepsy Behav.

[3] Vrinda M, Arun S, Srikumar BN, Kutty BM, Shankaranarayana Rao BS (2019). Temporal lobe epilepsy-induced neurodegeneration and cognitive deficits: Implications for aging. J Chem Neuroanat.

[4] Kanner AM, Helmstaedter C, Sadat-Hossieny Z, Meador K (2020). Cognitive disorders in epilepsy I: Clinical experience, real-world evidence and recommendations. Seizure.

[5] Springer C, Nappe TM. Anticonvulsants toxicity. StatPearls [Internet]. 2019. In: StatPearls [Internet]. Treasure Island (FL): StatPearls Publishing; 2022. 2021. PMID: 30725891 Bookshelf ID: NBK537206. 30725891

[6] Ru J, Li P, Wang J, Zhou W, Li B, Huang C (2014). TCMSP: a database of systems pharmacology for drug discovery from herbal medicines. J Cheminform.

[7] Naghizadeh A, Hamzeheian D, Akbari S, Mohammadi F, Otoufat T, Asgari S (2020). UNaProd: A Universal Natural Product Database for Materia Medica of Iranian Traditional Medicine. Evid-Based Complement Alternat Med.

[8] Aghili M (2014). Makhzan-al-Advieh.

[9] Nazem Jahan M. Exir-e A'zam (The Great Panacea). Tehran: Iran University of Medical Sciences, Institute of Medicine History, Islamic and Alternative Medicine; 2008. https://scholar.google.com/scholar_lookup?title=Exir-e+A%27zam+(The+Great+Panacea)&author=M+Nazem+Jahan&publication_year=2008&.

[10] Avicenna. Qanun Fi al-Teb (Canon of Medicine). Beirut: Dare Ehya al-Toras Institute; 2005.

[11] Cortés-Rojas DF, de Souza CRF, Oliveira WP (2014). Clove (Syzygium aromaticum): a precious spice. Asian Pac J Trop Biomed.

[12] Uddin MA, Shahinuzzaman M, Rana MS, Yaakob Z (2017). Study of chemical composition and medicinal properties of volatile oil from clove buds (Eugenia caryophyllus). Int J Pharm Sci Res.

[13] Said MM (2011). The protective effect of eugenol against gentamicin-induced nephrotoxicity and oxidative damage in rat kidney. Fundam Clin Pharmacol.

[14] Manikandan P, Vinothini G, Priyadarsini RV, Prathiba D, Nagini S (2011). Eugenol inhibits cell proliferation via NF-κB suppression in a rat model of gastric carcinogenesis induced by MNNG. Invest New Drugs.

[15] de Sá Júnior PL, Câmara DAD, Costa AS, Ruiz JLM, Levy D, Azevedo RA (2016). Apoptotic effect of eugenol envolves G2/M phase abrogation accompanied by mitochondrial damage and clastogenic effect on cancer cell in vitro. Phytomedicine.

[16] de Andrade FdCP, Mendes AN. Computational analysis of eugenol inhibitory activity in lipoxygenase and cyclooxygenase pathways. Sci Rep. 2020;10(1):16204. 10.1038/s41598-020-73203-z. 10.1038/s41598-020-73203-z PMC7530671 33004893

[17] Mesole SB, Alfred OO, Yusuf UA, Lukubi L, Ndhlovu D (2020). Apoptotic Inducement of Neuronal Cells by Aluminium Chloride and the Neuroprotective Effect of Eugenol in Wistar Rats. Oxid Med Cell Longev.

[18] Kabuto H, Tada M, Kohno M (2007). Eugenol [2-methoxy-4-(2-propenyl) phenol] prevents 6-hydroxydopamine-induced dopamine depression and lipid peroxidation inductivity in mouse striatum. Biol Pharm Bull.

[19] Moreira Vasconcelos CF, da Cunha Ferreira NM, Hardy Lima Pontes N, de Sousa Dos Reis TD, Basto Souza R, Aragão Catunda Junior FE, et al. Eugenol and its association with levodopa in 6-hydroxydopamine-induced hemiparkinsonian rats: Behavioural and neurochemical alterations. Basic Clin Pharmacol Toxicol. 2020;127(4):287–302. 10.1111/bcpt.13425. 10.1111/bcpt.13425 32353201

[20] Ma L, Mu Y, Zhang Z, Sun Q (2018). Eugenol promotes functional recovery and alleviates inflammation, oxidative stress, and neural apoptosis in a rat model of spinal cord injury. Restor Neurol Neurosci.

[21] Pezzoli M, Elhamdani A, Camacho S, Meystre J, González SM, Le Coutre J (2014). Dampened neural activity and abolition of epileptic-like activity in cortical slices by active ingredients of spices. Sci Rep.

[22] Avanthi E, Kumar LP, Somashekar H (2015). The study of antiepileptic activity of clove oil in chemical induced convulsions in mice. International Journal of Basic & Clinical Pharmacology.

[23] Joushi S, Salmani ME (2017). Effect of eugenol on lithium-pilocarpine model of epilepsy: Behavioral, histological, and molecular changes. Iran J Basic Med Sci.

[24] Kim SR (2016). Control of Granule Cell Dispersion by Natural Materials Such as Eugenol and Naringin: A Potential Therapeutic Strategy Against Temporal Lobe Epilepsy. J Med Food.

[25] Garabadu D, Shah A, Ahmad A, Joshi VB, Saxena B, Palit G (2011). Eugenol as an anti-stress agent: modulation of hypothalamic–pituitary–adrenal axis and brain monoaminergic systems in a rat model of stress. Stress.

[26] Mehta AK, Halder S, Khanna N, Tandon OP, Sharma KK (2013). The effect of the essential oil of Eugenia caryophyllata in animal models of depression and locomotor activity. Nutr Neurosci.

[27] Halder S, Mehta AK, Kar R, Mustafa M, Mediratta PK, Sharma KK (2011). Clove oil reverses learning and memory deficits in scopolamine-treated mice. Planta Med.

[28] Taheri P, Yaghmaei P, Tehrani HS, Ebrahim-Habibi A (2019). Effects of Eugenol on Alzheimer’s Disease-like Manifestations in Insulin-and Aβ-Induced Rat Models. Neurophysiology.

[29] Garabadu D, Sharma M (2019). Eugenol attenuates scopolamine-induced hippocampal cholinergic, glutamatergic, and mitochondrial toxicity in experimental rats. Neurotox Res.

[30] Pourgholami MH, Kamalinejad M, Javadi M, Majzoob S, Sayyah M (1999). Evaluation of the anticonvulsant activity of the essential oil of Eugenia caryophyllata in male mice. J Ethnopharmacol.

[31] Akbar L, Juliandi B, Boediono A, Batubara I, Subangkit M (2021). Effects of Eugenol on Memory Performance, Neurogenesis, and Dendritic Complexity of Neurons in Mice Analyzed by Behavioral Tests and Golgi Staining of Brain Tissue. J Stem Cells Regen Med.

[32] Kälviäinen R (2015). Intranasal therapies for acute seizures. Epilepsy Behav.

[33] Westin UE, Boström E, Gråsjö J, Hammarlund-Udenaes M, Björk E (2006). Direct nose-to-brain transfer of morphine after nasal administration to rats. Pharm Res.

[34] Bahrampouri S, Pakniyat A, Qaribi M, Habibzadeh Y (2020). Intranasal agents in the emergency care: A systematic review. Iranian Journal of Systematic Review in Medical Sciences.

[35] Racine RJ (1972). Modification of seizure activity by electrical stimulation: II. Motor seizure Electroencephalography and clinical neurophysiology.

[36] Le AP, Friedman WJ (2012). Matrix metalloproteinase-7 regulates cleavage of pro-nerve growth factor and is neuroprotective following kainic acid-induced seizures. J Neurosci.

[37] Hanson LR, Fine JM, Svitak AL, Faltesek KA. Intranasal administration of CNS therapeutics to awake mice. JoVE. 2013(74):e4440. 10.3791/4440. http://www.jove.com/video/4440. 10.3791/4440 PMC3653240 23608783

[38] Prut L, Belzung C (2003). The open field as a paradigm to measure the effects of drugs on anxiety-like behaviors: a review. Eur J Pharmacol.

[39] Hughes RN (2004). The value of spontaneous alternation behavior (SAB) as a test of retention in pharmacological investigations of memory. Neurosci Biobehav Rev.

[40] Rice AC, Floyd CL, Lyeth BG, Hamm RJ, DeLorenzo RJ (1998). Status epilepticus causes long-term NMDA receptor-dependent behavioral changes and cognitive deficits. Epilepsia.

[41] Paxinos G, Watson C. The rat brain in stereotaxic coordinates. 6th ed. San Diego: Elsevier Academic Press; 2006. https://0-scholar-google-com.brum.beds.ac.uk/citations?view_op=view_citation&hl=nl&user=9ddOX_wAAAAJ&citation_for_view=9ddOX_wAAAAJ:-jrNzM816MMC.

[42] Ramazi S, Fahanik-Babaei J, Mohamadi-Zarch S-M, Tashakori-Miyanroudi M, Nourabadi D, Nazari-Serenjeh M (2020). Neuroprotective and anticonvulsant effects of sinomenine in kainate rat model of temporal lobe epilepsy: Involvement of oxidative stress, inflammation and pyroptosis. J Chem Neuroanat.

[43] Cavalheiro E, Santos N, Priel M (1996). The pilocarpine model of epilepsy in mice. Epilepsia.

[44] Cavalheiro EA, Naffah-Mazzacoratti MG, Mello LE, Leite JP. The pilocarpine model of seizures. Models of seizures and epilepsy. 2006:433–48.

[45] Gröticke I, Hoffmann K, Löscher W (2007). Behavioral alterations in the pilocarpine model of temporal lobe epilepsy in mice. Exp Neurol.

[46] Engelborghs S (2000). D’hooge R, De Deyn P. Pathophysiology of epilepsy Acta neurologica belgica.

[47] Zavvari F, Mousavi SMM, Ejlali M, Barfi S, Karimzadeh F (2020). Glutamate Signaling Pathway in Absence Epilepsy: Possible Role of Ionotropic AMPA Glutamate Receptor Type 1 Subunit. Iranian Journal of Pharmaceutical Research: IJPR.

[48] Smolders I, Khan GM, Manil J, Ebinger G, Michotte Y (1997). NMDA receptor-mediated pilocarpine-induced seizures: characterization in freely moving rats by microdialysis. Br J Pharmacol.

[49] Houser CR, Esclapez M (1996). Vulnerability and plasticity of the GABA system in the pilocarpine model of spontaneous recurrent seizures. Epilepsy Res.

[50] Bahr TA, Rodriguez D, Beaumont C, Allred K (2019). The Effects of Various Essential Oils on Epilepsy and Acute Seizure: A Systematic Review. Evid Based Complement Alternat Med.

[51] da Fonsêca DV, da Silva Maia Bezerra Filho C, Lima TC, de Almeida RN, de Sousa DP. Anticonvulsant Essential Oils and Their Relationship with Oxidative Stress in Epilepsy. Biomolecules. 2019;9(12):835. 10.3390/biom9120835. 10.3390/biom9120835 PMC6995584 31817682

[52] Pourgholami M, Kamalinejad M, Javadi M, Majzoob S, Sayyah M (1999). Evaluation of the anticonvulsant activity of the essential oil of Eugenia caryophyllata in male mice. J Ethnopharmacol.

[53] Hosseini M, Jafarianheris T, Seddighi N, Parvaneh M, Ghorbani A, Sadeghnia HR (2012). Effects of different extracts of Eugenia caryophyllata on pentylenetetrazole-induced seizures in mice. Zhong Xi Yi Jie He Xue Bao.

[54] Chaieb K, Hajlaoui H, Zmantar T, Kahla‐Nakbi AB, Rouabhia M, Mahdouani K, et al. The chemical composition and biological activity of clove essential oil, Eugenia caryophyllata (Syzigium aromaticum L. Myrtaceae): a short review. Phytother Res. 2007;21(6):501–6. 10.1002/ptr.2124. 10.1002/ptr.2124 17380552

[55] Cho JS, Kim TH, Lim JM, Song JH (2008). Effects of eugenol on Na+ currents in rat dorsal root ganglion neurons. Brain Res.

[56] Lee SH, Moon JY, Jung SJ, Kang JG, Choi SP, Jang JH (2015). Eugenol inhibits the GABAA current in trigeminal ganglion neurons. PLoS ONE.

[57] Huang C-W, Chow JC, Tsai J-J, Wu S-N (2012). Characterizing the effects of Eugenol on neuronal ionic currents and hyperexcitability. Psychopharmacology.

[58] Gao XF, Liu Q, Qing H, Mu KM, Zhang J, Zhang D (2020). Development of eugenol-loaded submicron emulsion and its antiepileptic effect through regulating the oxidative stress. Int J Pharm.

[59] Dallmeier Zelger KR, Zelger JL, Carlini EA (1983). New anticonvulsants derived from 4-allyl-2-methoxyphenol (Eugenol): comparison with common antiepileptics in mice. Pharmacology.

[60] Vatanparast J, Khalili S, Naseh M (2017). Dual effects of eugenol on the neuronal excitability: An in vitro study. Neurotoxicology.

[61] Quintans-Júnior LJ, Guimarães AG, Araújo BE, Oliveira GF, Santana MT, Moreira FV (2010). Carvacrol, (-)-borneol and citral reduce convulsant activity in rodents. Afr J Biotech.

[62] Mishra RK, Baker MT (2014). Seizure prevention by the naturally occurring phenols, carvacrol and thymol in a partial seizure-psychomotor model. Bioorg Med Chem Lett.

[63] Khalil A, Kovac S, Morris G, Walker MC (2017). Carvacrol after status epilepticus (SE) prevents recurrent SE, early seizures, cell death, and cognitive decline. Epilepsia.

[64] Khokra S, Jain S, Prakash O (2011). Anticonvulsant activity of essential oils isolated from Vitex negundo Linn. Pharm Chem J.

[65] Savage K, Firth J, Stough C, Sarris J (2018). GABA-modulating phytomedicines for anxiety: A systematic review of preclinical and clinical evidence. Phytother Res.

[66] de Oliveira CC, de Oliveira CV, Grigoletto J, Ribeiro LR, Funck VR, Grauncke AC (2016). Anticonvulsant activity of β-caryophyllene against pentylenetetrazol-induced seizures. Epilepsy Behav.

[67] Tchekalarova J, da Conceição Machado K, Gomes Júnior AL, de Carvalho Melo Cavalcante AA, Momchilova A, Tzoneva R. Pharmacological characterization of the cannabinoid receptor 2 agonist, β-caryophyllene on seizure models in mice. Seizure. 2018;57:22–6. 10.1016/j.seizure.2018.03.009. 10.1016/j.seizure.2018.03.009 29547827

[68] Lou J, Cao G, Li R, Liu J, Dong Z, Xu L (2016). β-Caryophyllene Attenuates Focal Cerebral Ischemia-Reperfusion Injury by Nrf2/HO-1 Pathway in Rats. Neurochem Res.

[69] Viveros-Paredes JM, González-Castañeda RE, Gertsch J, Chaparro-Huerta V, López-Roa RI, Vázquez-Valls E, et al. Neuroprotective Effects of β-Caryophyllene against Dopaminergic Neuron Injury in a Murine Model of Parkinson's Disease Induced by MPTP. Pharmaceuticals. 2017;10(3):60. 10.3390/ph10030060. 10.3390/ph10030060 PMC5620604 28684694

[70] Yang M, Lv Y, Tian X, Lou J, An R, Zhang Q, et al. Neuroprotective Effect of β-Caryophyllene on Cerebral Ischemia-Reperfusion Injury via Regulation of Necroptotic Neuronal Death and Inflammation: In Vivo and in Vitro. Front Neurosci. 2017;11:583. 10.3389/fnins.2017.00583. 10.3389/fnins.2017.00583 PMC5662640 29123466

[71] Dixon Clarke SE, Ramsay RR (2011). Dietary inhibitors of monoamine oxidase A. J Neural Transm (Vienna).

[72] Mehta AK, Halder S, Khanna N, Tandon OP, Sharma KK (2013). The effect of the essential oil of Eugenia caryophyllata in animal models of depression and locomotor activity. Nutr Neurosci.

[73] Wang X, Chen Y, Wang Q, Sun L, Li G, Zhang C, et al. Support for Natural Small-Molecule Phenols as Anxiolytics. Molecules. 2017;22(12):2138. 10.3390/molecules22122138. 10.3390/molecules22122138 PMC6150002 29210995

[74] Siyal FJ, Memon Z, Siddiqui RA, Aslam Z, Nisar U, Imad R, et al. Eugenol and liposome-based nanocarriers loaded with eugenol protect against anxiolytic disorder via down regulation of neurokinin-1 receptors in mice. Pak J Pharm Sci. 2020;33(5(Supplementary)):2275–84. PMID: 33832901. 33832901

[75] Halder S, Mehta AK, Mediratta PK, Sharma KK (2012). Acute effect of essential oil of Eugenia caryophyllata on cognition and pain in mice. Naunyn Schmiedebergs Arch Pharmacol.

[76] Halder S, Mehta AK, Kar R, Mustafa M, Mediratta PK, Sharma KK (2011). Clove oil reverses learning and memory deficits in scopolamine-treated mice. Planta Med.

[77] Liu Z, Niu W, Yang X, Wang Y (2013). Effects of combined acupuncture and eugenol on learning-memory ability and antioxidation system of hippocampus in Alzheimer disease rats via olfactory system stimulation. J Tradit Chin Med.

[78] Garabadu D, Sharma M (2019). Eugenol Attenuates Scopolamine-Induced Hippocampal Cholinergic, Glutamatergic, and Mitochondrial Toxicity in Experimental Rats. Neurotox Res.

[79] Barot J, Saxena B (2021). Therapeutic effects of eugenol in a rat model of traumatic brain injury: A behavioral, biochemical, and histological study. J Tradit Complement Med.

[80] Ma L, Mu Y, Zhang Z, Sun Q (2018). Eugenol promotes functional recovery and alleviates inflammation, oxidative stress, and neural apoptosis in a rat model of spinal cord injury. Restor Neurol Neurosci.

[81] Keller L-A, Merkel O, Popp A (2022). Intranasal drug delivery: opportunities and toxicologic challenges during drug development. Drug Deliv Transl Res..

[82] Pires A, Fortuna A, Alves G, Falcão A (2009). Intranasal drug delivery: how, why and what for?. J Pharm Pharm Sci.

[83] Ozgoli G, Torkashvand S, Moghaddam FS, Borumandnia N, Mojab F, Minooee S (2016). Comparison of peppermint and clove essential oil aroma on pain intensity and anxiety at first stage of labor. Iranian Journal of Obstetrics, Gynecology and Infertility.

[84] Ekinci Akdemir FN, Yildirim S, Kandemir FM, Aksu EH, Guler MC, Kiziltunc Ozmen H (2019). The antiapoptotic and antioxidant effects of eugenol against cisplatin-induced testicular damage in the experimental model. Andrologia.

[85] Magalhães CB, Casquilho NV, Machado MN, Riva DR, Travassos LH, Leal-Cardoso JH (2019). The anti-inflammatory and anti-oxidative actions of eugenol improve lipopolysaccharide-induced lung injury. Respir Physiol Neurobiol.

[86] Irie Y (2006). Effects of eugenol on the central nervous system: its possible application to treatment of Alzheimer's disease, depression, and Parkinson's disease. Curr Bioact Compd.

